# Assemblages of pelagic thaliaceans in oceanographic features at the tropical-temperate transition zone of a western boundary current

**DOI:** 10.1093/plankt/fbad024

**Published:** 2023-06-02

**Authors:** Kylie A Pitt, Jonathan W Lawley, Charles Hinchliffe, Paloma A Matis, Carolina OlguÍn-Jacobson, Nur Arafeh-Dalmau, Pauline Lindholm, Jade Arnold, Iain M Suthers

**Affiliations:** School of Environment and Science, Coastal and Marine Research Centre, Australian Rivers Institute, Gold Coast Campus, Griffith University, Southport, QLD 4222, Australia; School of Environment and Science, Coastal and Marine Research Centre, Australian Rivers Institute, Gold Coast Campus, Griffith University, Southport, QLD 4222, Australia; School of Biological, Earth, and Environmental Sciences, University of New South Wales, Sydney, NSW 2052, Australia; School of Biological, Earth, and Environmental Sciences, University of New South Wales, Sydney, NSW 2052, Australia; Sydney Institute of Marine Science, Chowder Bay Road, Mosman, NSW 2088, Australia; School of Environment and Science, Coastal and Marine Research Centre, Australian Rivers Institute, Gold Coast Campus, Griffith University, Southport, QLD 4222, Australia; Hopkins Marine Station, Stanford Doerr School of Sustainability, Stanford University, Pacific Grove, Stanford, CA 94305, USA; Hopkins Marine Station, Stanford Doerr School of Sustainability, Stanford University, Pacific Grove, Stanford, CA 94305, USA; Centre for Biodiversity and Conservation Science, School of Biological Sciences, The University of Queensland, St Lucia, QLD 4072, Australia; Department of Geography, University of California Los Angeles, Los Angeles, CA 90095, USA; School of Environment and Science, Coastal and Marine Research Centre, Australian Rivers Institute, Gold Coast Campus, Griffith University, Southport, QLD 4222, Australia; School of Environment and Science, Coastal and Marine Research Centre, Australian Rivers Institute, Gold Coast Campus, Griffith University, Southport, QLD 4222, Australia; School of Biological, Earth, and Environmental Sciences, University of New South Wales, Sydney, NSW 2052, Australia; Sydney Institute of Marine Science, Chowder Bay Road, Mosman, NSW 2088, Australia

**Keywords:** thaliacean communities, East Australian Current, cyclonic eddies, pyrosomes, doliolids, salps

## Abstract

Mesoscale oceanographic features influence the composition of zooplankton. Cyclonic eddies can promote upwelling and production of gelatinous zooplankton, which play critical roles in ocean biogeochemical cycling. We examined variation in assemblages of thaliaceans (salps, doliolids and pyrosomes) among mesoscale oceanographic features at the tropical-temperate boundary of the East Australian Current (EAC) in Spring 2019 and Autumn 2021. The influence of cyclonic eddies was examined in a large offshore cyclonic eddy in 2019 and a newly formed frontal eddy in 2021. Pyrosomes were most abundant in the offshore EAC jet, and salps and doliolids were most abundant in coastal features, including within eddies that were transported offshore. In 2019, *Salpa fusiformis* increased 4-fold over 8 days in the large cyclonic eddy, and in 2021, doliolids increased > 50-fold over 2 weeks in a chlorophyll-rich coastal eddy while abundances of other thaliaceans remained unchanged or decreased. Correlations between abundances of thaliaceans and chlorophyll-a concentrations across the 102 samples collected during both voyages revealed that doliolids occupy a wider range of chlorophyll-a concentrations than salps. Our observations indicate that doliolids thrive in productive shelf environments, salps occur in less productive shelf waters and pyrosomes are most abundant in oligotrophic waters of the south Coral Sea.

## INTRODUCTION

Gelatinous zooplankton comprise diverse taxa including cnidarian jellyfishes, ctenophores, pelagic mollusks and thaliaceans (Chordata), commonly known as salps, doliolids and pyrosomes. Thaliaceans often comprise a large component of the pelagic zooplankton biomass and frequently dominate the zooplankton assemblages of coastal and global oceans (Lucas *et al.,* 2014). They are a significant and nutritious food source for a diverse range of pelagic predators including turtles (Dodge *et al.,* 2011), penguins (Cavallo *et al.,* 2018) and fish (some of which are commercially harvested; Henschke *et al.,* 2016) and are a critical component of the biological pump (Condon *et al.,* 2011; Luo *et al.,* 2020). Indeed, thaliaceans may contribute to > 30% of global particulate organic carbon export through sinking of their fecal pellets and carcasses (Luo *et al.,* 2020). The unique, watery body plans of thaliaceans facilitate high clearance and growth rates (Acuna *et al.,* 2011; Pitt *et al.,* 2013) and their extraordinary rates of sexual and asexual reproduction enable populations of some species to double within hours to days (Deibel and Lowen, 2012). These characteristics enable populations to respond quickly to changes in primary production. Knowledge of the dynamics and drivers of thaliacean populations is thus necessary for understanding pelagic food webs and global biogeochemical cycles.

Oceanographic features such as cyclonic eddies that promote upwelling and frontal eddies that entrain nutrient rich shelf waters (Everett *et al.,* 2015) may drive regional-scale increases in thaliaceans. For example, the pyrosome, *Pyrosoma atlanticum,* was two-orders of magnitude more abundant in a cold core (i.e. upwelling) eddy than in warm core eddies in the Tasman Sea (Henschke *et al.,* 2019), and the greatest concentration of salps ever recorded (the species *Thalia democratica*), similarly occurred in a cyclonic frontal eddy (FE) of the East Australian Current (EAC; Everett *et al.,* 2011). Concentrations of the doliolid, *Dolioletta gegenbauri*, also exceeded 3 000 m^−3^ within a cold core eddy that originated in the Gulf Stream and intruded upon the continental shelf of Florida (Deibel, 1985).

Western boundary currents (WBCs) are the most energetic currents in the ocean. They transport warm, tropical waters polewards and strongly influence regional climate (Sprintall *et al.,* 1995) and fisheries (Hofmann and Powell, 1998; Suthers *et al.,* 2011; Young *et al.,* 2011). Although the poleward jet of a WBC is the dominant feature, current meanders, local topography and changes in current velocities and trajectories produce distinct meso-scale oceanographic features. These include cyclonic (upwelling) and anti-cyclonic (downwelling) eddies and small cyclonic frontal eddies that form through instabilities or when the coastal edge of a WBC intercepts prominent headlands (Ismail *et al.,* 2017). Acceleration of flow and divergence of water masses can also generate regions of upwelled, cool and nutrient rich water (e.g. Everett *et al.,* 2014). These different mesoscale features strongly influence primary production (Everett *et al.,* 2014), support different biological communities and may have a major influence on regional plankton dynamics (Henschke *et al.,* 2015), including the production of thaliaceans (e.g. Pagés *et al.,* 2001; Riandey *et al.,* 2005).

**Fig. 1:**
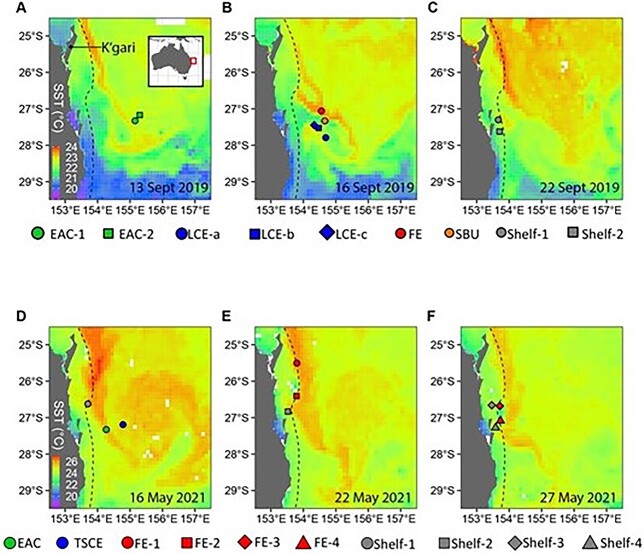
SST maps indicating the location of the stations sampled in the intensification zone of the EAC during Spring 2019 (top) and Autumn 2021 (bottom) voyages. Stations are represented as the mean of the latitude and longitude of the replicate tows within each station. Symbol colors indicate the type of oceanographic feature and symbol shapes indicate replicate stations within each type of oceanographic feature. K’gari (formally Fraser Island) is indicated in 1A. EAC = East Australia Current; LCE = large cyclonic eddy (sampled three times over 8 days; LCE-a, LCE-b, LCE-c); FE = frontal eddy; SBU = shelf break upwelling; TSCE = Tasman Sea cyclonic eddy.

The EAC is the WBC of the South Pacific Gyre and is the dominant oceanographic feature along Australia’s east coast (Suthers *et al.,* 2011). The EAC originates between 15°S and 20°S from the southern bifurcation of the South Equatorial Current (Ridgway and Dunn, 2003). The current nears the coast and intensifies as it flows around the most easterly section of the Australian continent between 23°S and 31°S. During Spring and Summer, periodic upwelling occurs, associated with current-driven bottom stress (referred to as the Southeast Fraser Island Upwelling System; Brevia *et al.,* 2015), creating a marine ecological hotspot (Ribbe and Brieva, 2016). The region within 100 km of the coast is dominated by short-lived (7–28 days) eddies with over 40% of all short-lived eddies in the EAC occurring in this region (Ribbe and Brieva, 2016). Smaller cyclonic frontal eddies also form when the western edge of the EAC intercepts prominent headlands on K’gari (formally Fraser Island; Fig. 1) at 25° S (Ribbe *et al.,* 2018). Mesoscale cyclonic eddies can entrain coastal water and generate cross-shelf transport and near-shore northward flows as a sporadic counter current to the EAC (Brieva *et al.,* 2015; Ribbe and Brieva, 2016). South of the intensification zone, the EAC slows and separates into easterly and southerly flows. Complex fields of cyclonic and anti-cyclonic eddies are generated south of the separation zone (Everett *et al.,* 2012; Ridgway and Dunn, 2003) and support production and cross shelf transport of commercially important fish larvae (e.g. Malan *et al.,* 2020; Matis *et al.,* 2014; Mullaney *et al.,* 2011) and massive blooms of pyrosomes (Henschke *et al.,* 2019) and salps (Everett *et al.,* 2011).

Although the EAC south of the separation zone has been reasonably well studied, the biology of the northern intensification zone has received much less attention. This is surprising, as this region at the tropical/temperate boundary is at the forefront of range expansions by tropical species e.g. (Armbrecht *et al.,* 2015; Verges *et al.,* 2016) and sustains important pelagic fisheries (Revill *et al.,* 2009; Young *et al.,* 2011). Studies of planktonic communities in the intensification zone have been limited to shelf waters, including studying changes in phytoplankton communities among seasons (Armbrecht *et al.,* 2015; Ribbe and Brieva, 2016) and during upwelling and downwelling events (Armbrecht *et al.,* 2014).

This study aimed to understand the relationship between mesoscale oceanographic features and assemblages of thaliaceans in the intensification zone of the EAC during the austral Spring in 2019 and the austral Autumn in 2021. In addition to sampling the prominent oceanographic features present during each voyage, the specific role of mesoscale eddies in facilitating production of thaliaceans was studied by sampling a large, offshore cyclonic eddy three times over 8 days in 2019 and sampling a FE four times over 15 days in 2021. We hypothesized that different mesoscale features would host different thaliacean assemblages and that cyclonic eddies would support production of thaliaceans.

## METHODS

### Study area

The study focused on the central region of the intensification zone in southeast Queensland (between 25.388° S and 27.955° S and 155.341° E and 153.462° E) where the EAC is most coherent (Fig. 1). The study area coincided with an oceanographic mooring array that monitors the dynamics of the EAC and extends across the continental shelf to the abyssal plain at 27° S (Sloyan *et al.,* 2016).

### Sampling of thaliaceans

Thaliaceans were sampled during the austral Spring (11–27 September 2019) and austral Autumn (14 May to 2 June 2 2021) during voyages on *RV Investigator*. Some thaliaceans (including pyrosomes (Andersen and Sardou, 1994; Henschke *et al.,* 2019) and some salps (e.g. *Salpa fusiformis*; e.g. Pascual *et al.,* 2017) undertake diel vertical migration (DVM), and migrating taxa are typically more abundant in shallow waters at night. To minimize potential confounding effects of DVM, thaliaceans were sampled only at night. Sampling commenced at least 1 hour after sunset and concluded more than one hour before sunrise. In 2019, 5–10 replicate plankton samples were collected at nine stations within five different oceanographic features (Table 1; Fig. 1), including two stations within the EAC jet (Fig. 1a); within a transient shelf break upwelling zone (Fig. 1b), within two cyclonic eddies [one FE was sampled once and the second large cyclonic eddy (LCE) was sampled three times over 8 days (Fig. 1b)], and two stations over the inshore continental shelf (Fig. 1c). In 2021, four replicate plankton samples were collected within four types of oceanographic features including one station in the EAC jet (Fig. 1d), one station in an offshore Tasman Sea cyclonic eddy (TSCE, Fig. 1d), four stations over the inshore continental shelf and four stations within a FE along the edge of the shelf (Fig. 1d, f). The FE was first sampled ~ 4 days after it formed and was last sampled 14 days later as the eddy decayed.

**Table 1:** Sampling details and hydrographic characteristics of the oceanographic features sampled during 2019 and 2021. Temperature, salinity and chlorophyll values were sampled from the underway seawater at a depth of 4 m. Values are mean ± SD. *N* = number of net tows, Temp = temperature

	Oceanographic feature	Date sampled	*N*	Temp (°C)	Salinity	Chl-a (μg L^−1^)	Bathymetric depth (m)
May 2019	EAC-1	11/9/2019	7	21.82 ± 0.03	35.66 ± 0.01	1.26 ± 0.17	4 772 ± 19
	EAC-2	13/9/2013	7	21.64 ± 0.06	35.68 ± 0.01	0.62 ± 0.21	4 774 ± 26
	Large Cyclonic Eddy-1	15/9/2019	10	21.17 ± 0.30	35.60 ± 0.01	0.71 ± 0.30	4 048 ± 681
	Large Cyclonic Eddy-2	20/9/2019	6	21.62 ± 0.21	35.60 ± 0.01	0.36 ± 0.13	3 404 ± 236
	Large Cyclonic Eddy-3	23/9/2019	6	21.78 ± 0.16	35.61 ± 0.03	0.13 ± 0.03	3 566 ± 82
	Shelf break upwelling	16/9/2019	6	20.50 ± 0.09	35.72 ± 0.01	1.24 ± 0.33	4 781 ± 3
	Small frontal eddy	18/9/2019	6	21.24 ± 0.10	35.59 ± 0.01	0.60 ± 0.12	4 813 ± 29
	Shelf-1	22/9/2019	8	21.66 ± 0.21	35.62 ± 0.01	0.33 ± 0.14	122 ± 5
	Shelf-2	26 and 27/9/2019	6	21.39 ± 0.26	35.64 ± 0.01	1.22 ± 0.35	86 ± 1
September 2021	Tasman Sea cyclonic eddy	14/5/2021	4	24.69 ± 0.02	35.29 ± 0.00	0.03 ± 0.02	4 179 ± 112
	EAC Frontal Eddy-1	16/5/2021 19/5/2021	4 4	25.19 ± 0.07 22.29 ± 0.05	35.22 ± 0.00 35.44 ± 0.008	0.14 ± 0.05 5.94 ± 0.54	4 772 ± 1 237 ± 16
	Frontal Eddy-2	23/5/2021	4	21.47 ± 0.21	35.53 ± 0.02	6.34 ± 0.37	136 ± 3
	Frontal Eddy-3	26/5/2021	4	22.15 ± 0.05	35.44 ± 0.01	1.63 ± 0.12	367 ± 66
	Frontal Eddy-4	2/6/2021	3	22.27 ± 0.91	35.41 ± 0.005	1.27 ± 0.31	231 ± 1.15
	Shelf-1	19/5/2021	4	24.05 ± 0.13	35.25 ± 0.01	0.26 ± 0.11	263 ± 20
	Shelf-2	25/5/2021	4	24.3 ± 35.23	35.23 ± 0.04	0.25 ± 0.02	92 ± 11
	Shelf-3	26/5/2021	4	24.19 ± 0.01	35.22 ± 0.00	0.14 ± 0.03	63 ± 3
	Shelf-4	27/5/2021	4	22.28 ± 0.09	35.42 ± 0.01	0.17 ± 0.05	78 ± 2

Thaliaceans were sampled using a bongo net (mouth diameter: 0.7 m; mesh size: 500 μm) that was towed obliquely and fitted with a mechanical flow meter (General Oceanics, Florida, USA). In 2019, the average maximum depth attained by the net was 43 m (± 7.1 m SD), the durations of the tows were 13–19 minutes (mean = 16), the speed of the tow ranged from 1 to 1.5 m s^−1^, and the average volume sampled was 693 m^3^ (± 256 m^3^ SD). In 2021, the average maximum depth sampled was 27 m (± 5.9 m SD), the tow duration ranged from 9 to 16 minutes (mean = 11), the tow speed ranged from 1 to 1.5 ms^−1^ and the average volume sampled was 673 m^3^ (± 293 m^3^ SD). Shorter (and so slightly shallower) tows were done in 2021 to reduce the volume of material sampled. Samples were immediately preserved in 5% formalin.

Thaliaceans were separated from the remaining zooplankton and identified and counted. Aggregate and solitary forms of salps were counted separately and pyrosomes were counted as entire colonies. Thaliaceans were identified to species, except for doliolids, which were aggregated into Order Doliolida because many were damaged and could not be identified to narrower taxonomic categories. All life stages of Doliolida were counted, and although it was not possible to consistently identify species, *Dolioletta gegenbauri* were common. Abundances were standardized to concentrations (individuals m^−3^). An index of production of the most abundant salp, *S. fusiformis,* was created using the ratio of aggregate to solitary zooids, based on the expectation that a greater proportion of aggregates would occur in a rapidly growing population (Foxton, 1966; Henschke and Pakhomov, 2019).

### Sampling of zooplankton for laser optical plankton counter analysis

Zooplankton were sampled using a 100-μm mesh, 0.2-m diameter ring net that was mounted inside the bongo net. Zooplankton were immediately fixed in 5% formalin and counted in the laboratory using a Laser Optical Plankton Counter (LOPC, Rolls Royce Canada Ltd, Canada) that counted particles and aggregates ranging in size from 200 to 30 000 μm (Herman *et al.,* 2004). In Autumn 2021, dense concentrations of the cyanobacterium *Trichodesmium* in the surface waters at the fourth a FE station overwhelmed the LOPC and resulted in average concentrations of particles ~ 15–50 times greater than at other locations. LOPC data from this station, therefore, were excluded from analysis.

### Physical and biological oceanography

Temperature (°C), salinity and fluorescence (dimensionless) were recorded during each bongo net tow using a Sea-Bird-SBE 21 thermosalinograph (Sea-Bird Scientific, USA) and fluorometer (WETStar, WETLabs, USA) that sampled underway seawater collected from a drop keel 4 m below the hull of the RV Investigator at 1 Hz. During the 2021 voyage, chl-a was sampled 10 times at night across a range of fluorescence readings and used to convert fluorescence readings to chl-a concentrations. Two liters of water were collected from the underway seawater system and vacuum filtered through GF/F filters. Filter papers were maintained in darkness and frozen until analyzed using a standard acetone extraction fluorescence method (*sensu*  Strutton *et al.,* 2011). The relationship between chl-a and fluorescence was established using a linear regression (chl-a = 0.6902 × fluorescence; *R*^2^ = 0.92; Supplementary Fig. 1). Bathymetric depths of each tow were derived values from GEBCO 15 arcsecond Grid (IOC *et al.,* 2003). All instruments were calibrated at the CSIRO Calibration Facility in Hobart.

### Statistical analyses

Data from each voyage were analyzed separately because different types of oceanographic features were sampled. Variation in assemblages of thaliaceans among oceanographic features was analyzed using one-way nonparametric permutational multivariate analyses of variance (PERMANOVA); (Anderson, 2001). Data were square-root-transformed and the resemblance matrices constructed using Bray–Curtis similarity. Pair-wise comparisons were used to identify which features differed. Differences in assemblages among oceanographic features were visualized using canonical analysis of principal coordinates (CAP; Anderson and Willis, 2003). The number of axes (*m*) was selected to minimize the leave-one-out residual sum of squares. The dominant species responsible for differences among features were identified based on Pearson’s correlations (*R* values > 0.4).

Univariate analyses of concentrations of total thaliaceans, taxon richness, total zooplankton (as measured using the LOPC) and individual species of thaliaceans highlighted by CAP as driving differences among oceanographic features were analyzed using one-way PERMANOVAs on square-root-transformed data. Resemblance matrices were constructed using the zero-adjusted Bray–Curtis dissimilarity metric (Clarke *et al.,* 2006). When significant differences were identified, pair-wise PERMANOVAs were used to identify which features differed.

## RESULTS

### Regional oceanography

In Spring 2019, a LCE dominated the region (Fig. 1a–c). The LCE formed at the edge of the shelf from the easterly divergence of the EAC approximately a month before being sampled and moved offshore. Temperature increased by > 0.5°C and the chl-a concentration decreased 5.4-fold over the three times (8 days) the LCE was sampled (Table 1). At the start of the voyage, the warm EAC jet was situated offshore (Fig. 1a) and concentrations of chl-a at EAC-1 were double those at EAC-2 (Table 1). A smaller cyclonic FE formed on approximately 12 September 2019, 6 days before it was sampled, when the western edge of the EAC intercepted K’gari. The FE entrained shelf water (Fig. 1b) and was characterized by intermediate values of temperature, salinity and chl-a (Table 1). The shelf break upwelling was a transient feature that formed 1–2 days before being sampled, driven by the eastward flow of the EAC to the north and the eastward flow of the LCE to the south (Fig. 1b). It was 0.6–1.1°C cooler and more saline than any other feature sampled, with relatively high values of chl-a, indicative of upwelling (Table 1).

At the end of the voyage, shelf waters were sampled twice and both occasions coincided with a transient incursion of the EAC onto the shelf, as characterized by temperatures at the shelf locations being ~ 0.5°C warmer at the end of the voyage compared to the beginning (Fig. 1c). CTD casts from RV Investigator indicated the EAC water occupied the upper 25 m of the water column, with a sharp thermocline demarcating the EAC water from the cooler shelf water below. The SST was ~ 0.3°C cooler and chl-a concentrations were marginally higher at Shelf-2 than at Shelf-1 indicating that the EAC incursion may have been greater at Shelf-1 (Table 1).

In Autumn 2021, a LCE was located on the eastern side of the EAC in the Tasman Sea (Fig. 1d). The warm EAC jet was initially located offshore but approached the coast and by the end of the voyage impinged on the continental shelf (Fig. 1d–f). A FE began to form adjacent to K’gari, on the eastern edge of the continental shelf on 15–16 May 2021. The FE was ~ 1.5°C cooler than the shelf water to the west and > 3° cooler than the EAC jet to the east indicating upwelling of cool water from the shelf break (Fig. 1e). During the first two times sampled (19 and 23 May 2021), concentrations of chl-a within the FE exceeded 5 μg L^−1^ and were > 20 greater than in the adjacent shelf water (Table 1; Supplementary Fig. 2) and > 5-fold of the largest concentrations observed during Spring 2019. The eddy disappeared 1–2 days after was last sampled as the EAC impinged the coast.

### Relationship between thaliaceans and oceanographic features

**Fig. 2:**
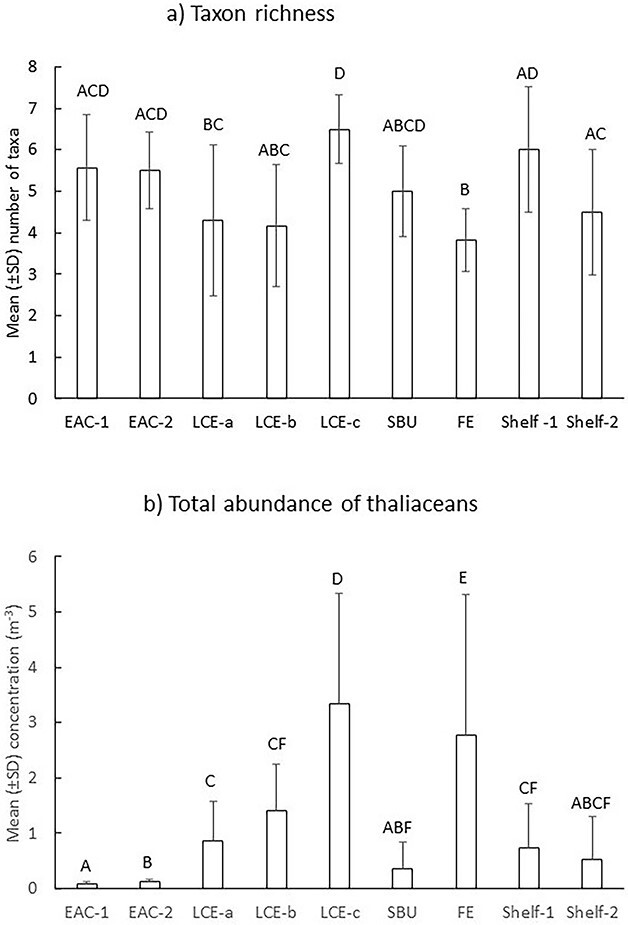
Spring 2019. Mean (±SD) taxon richness (**a**) and total abundances (**b**) of thaliaceans among oceanographic features. EAC = East Australia Current; LCE = large cyclonic eddy (sampled three times over 8 days; LCE-a, LCE-b, LCE-c); SBU = shelf break upwelling; FE = frontal eddy. Letters above bars indicate similarity (e.g. AA) or differences (e.g. AB) between oceanographic features and stations.

In Spring 2019, 12 taxa of thaliaceans were sampled (Supplementary Table 1). Catches were dominated by *S. fusiformis* (67%), Doliolida (26%) and *T. democratica* (4.5%). Taxon richness varied among stations (Pseudo-F = 3.0; *P* = < 0.01) with fewest taxa occurring in the FE and the LCE during the first time sampled (Fig. 2a). Overall concentrations of thaliaceans also varied (Pseudo-F = 6.8; *P* = < 0.01). Concentrations were smallest in the EAC jet, shelf waters and the shelf break upwelling and greatest in the FE and the LCE during the third time sampled (Fig. 2b).

**Fig. 3:**
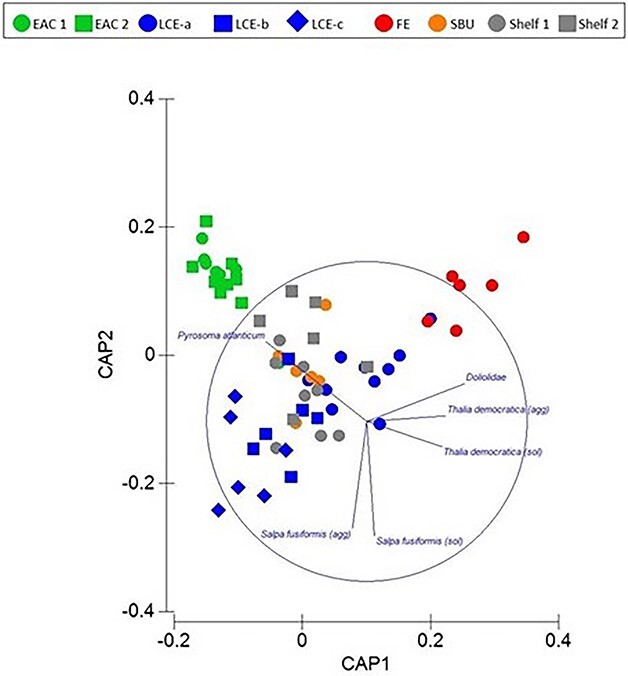
Spring 2019. Constrained CAP analysis illustrating relationships among thaliaceans and oceanographic features and stations (δ2 = 0.8835; *m* = 7; LoA = 60.317%). EAC = East Australia Current; LCE = large cyclonic eddy; SBU = shelf break upwelling; FE = frontal eddy.

Assemblages of thaliaceans varied among oceanographic features (PERMANOVA Pseudo-F = 6.8079; *P* = 0.001; Fig. 3). Pairwise comparisons revealed that assemblages differed between the three times the LCE was sampled. Assemblages did not differ between the two EAC stations nor between the two shelf stations, and the shelf break upwelling did not differ from either of the shelf stations or the LCE during the second time sampled. Assemblages within the FE differed to all other oceanographic features (Fig. 3).

**Fig. 4:**
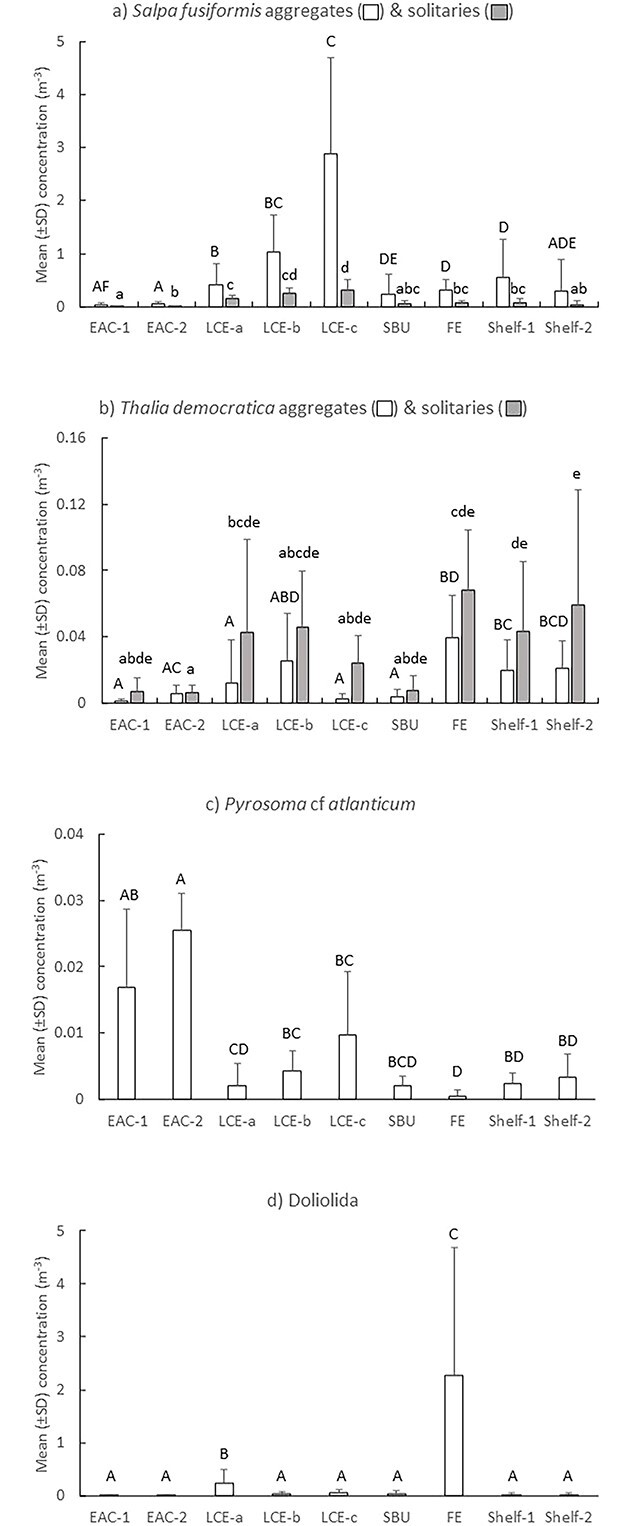
Spring 2019. Mean (±SD) concentrations of *S. fusiformis* (**a**), *T. democratica* (**b**), *Pyrosoma* cf.* atlanticum* (**c**), and Doliolida (**d**) among oceanographic features and stations. EAC = East Australia Current; LCE = large cyclonic eddy (sampled three times over 8 days; LCE-a, LCE-b, LCE-c); SBU = shelf break upwelling; FE = frontal eddy. Letters above bars indicate similarity (e.g. AA) or differences (e.g. AB) between oceanographic features for aggregates (capital letters) and solitaries (lowercase letters). Note different scales on *y*-axes.

The dissimilarity in thaliacean assemblages among oceanographic features was mainly caused by differing relative abundances of aggregate and solitary forms of *S. fusiformis* and *T. democratica*, Doliolida and *Pyrosoma* cf. *atlanticum* (Fig. 3; Table 2). In the LCE, aggregates of *S. fusiformis* increased 6-fold and concentrations of solitaries doubled over the 8 days the eddy was sampled (Fig. 4a). By the last time sampled, concentrations of *S. fusiformis* aggregates in the LCE were more than five times greater than in any other oceanographic feature. Aggregates and solitaries of *S. fusiformis* were least abundant in the EAC stations and solitaries were also scarce in the shelf break upwelling, the FE and over the shelf. Concentrations of *T. democratica* were highly variable and solitaries were more common than aggregates in most oceanographic features (Fig. 4b). Solitaries were most concentrated in shelf waters, within the FE, and during the first two times the LCE was sampled. Like *S. fusiformis*, aggregates and solitaries of *T. democratica* were scarce in the EAC jet and shelf break upwelling. *Pyrosoma* cf. *atlanticum* tended to be most abundant in the EAC jet (Fig. 4c), whereas Doliolida were only abundant in the FE (Fig. 4d).

**Table 2:** Results of one-way PERMANOVAs comparing concentrations of individual taxa among oceanographic features

Voyage	Taxon	Pseudo-F	*P*
May 2019	*Salpa fusiformis* (aggregate)	4.1325	0.001
	*Salpa fusiformis* (solitary)	4.2173	0.001
	*Thalia democratica* (aggregate)	3.4065	0.003
	*Thalia democratica* (solitary)	2.1915	0.013
	*Traustedtia multitentaculata* (aggregate)	3.9536	0.002
	Order Doliolida	3.1026	0.005
	*Soestia zonaria* (aggregate)	3.6636	0.001
	*Pyrosoma* cf *atlanticum* colonies	3.0247	0.005
September 2021	*Salpa fusiformis* (aggregate)	3.2825	0.003
	*Salpa fusiformis* (solitary)	5.1906	0.004
	*Thalia democratica* (aggregate)	2.2242	0.019
	*Thalia democratica* (solitary)	3.5820	0.003
	*Thalia rhomboides* (aggregate)	14.812	0.001
	*Thalia rhomboides* (solitary)	3.8001	0.005
	Order Doliolida	12.441	0.001

**Fig. 5:**
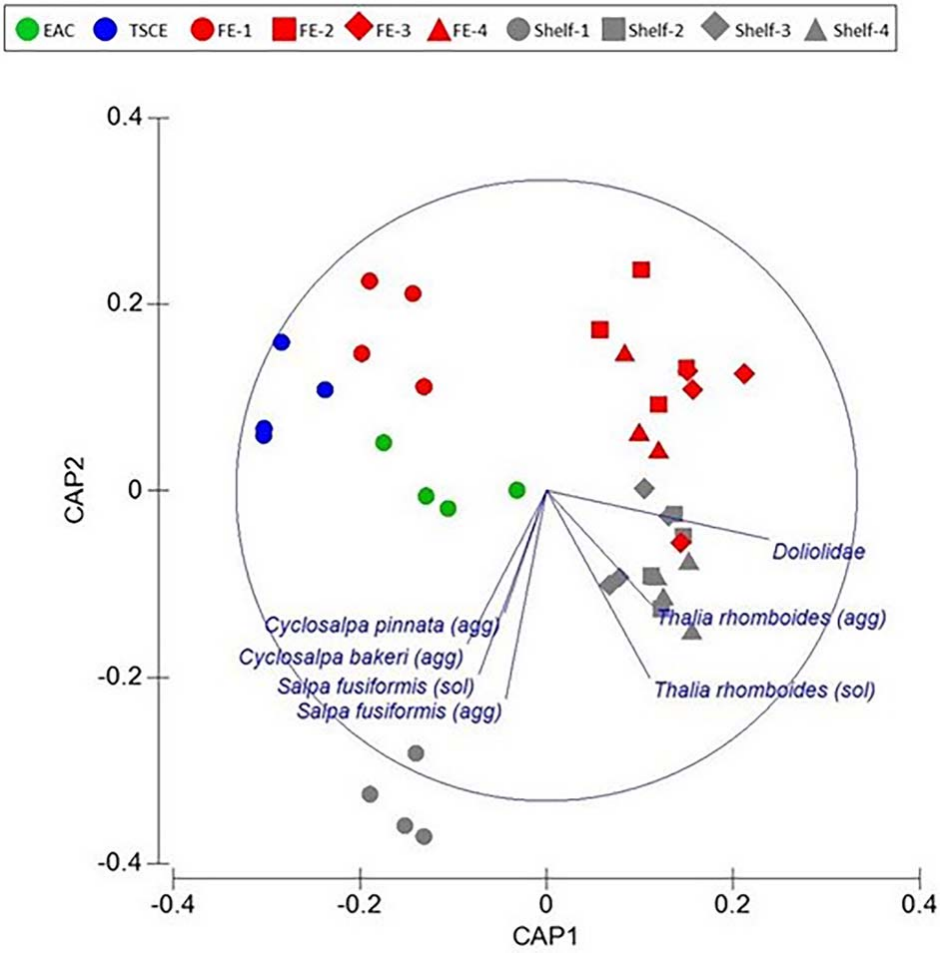
Autumn 2021. Constrained CAP analysis illustrating relationships among thaliaceans and oceanographic features and stations (δ2 = 0.9678; *m* = 6; LoA = 71.795%). EAC = East Australia Current; TSCE = Tasman Sea cyclonic eddy; FE = frontal eddy.

In Autumn 2021, eight taxa of thaliaceans were sampled (Supplementary Table 1). Doliolida comprised 87% of the catch. *Thalia rhomboides*, which was absent in Spring 2019, contributed to 8% of the catch and *T. democratica* comprised 3%. *Salpa fusiformis* comprised < 1% of the catch. Taxon richness did not vary among stations (Pseudo-F = 1.3; *P* = 0.284) but assemblages of thaliaceans differed among all oceanographic features (Pseudo-F = 12.91; *P* < 0.01; Fig. 5). Assemblages remained similar during the last three times the FE was sampled and were also similar during the second and fourth times the shelf waters were sampled. Differences in thaliacean assemblages among oceanographic features were largely caused by differing abundances of Doliolida and aggregate and solitary stages of the salps *Thalia rhomboides* and *S. fusiformis* (Fig. 5; Table 2). *Thalia rhomboides* and *S. fusiformis* were strongly associated with shelf waters whilst Doliolida were associated with both shelf and FE stations. Aggregate stages of *Cyclosalpa pinnata* and *C. bakeri* also drove some of the differences between oceanographic features but these species only occurred at the first shelf location in very small concentrations (<0.2 individuals m^−3^; Fig. 5).

**Fig. 6:**
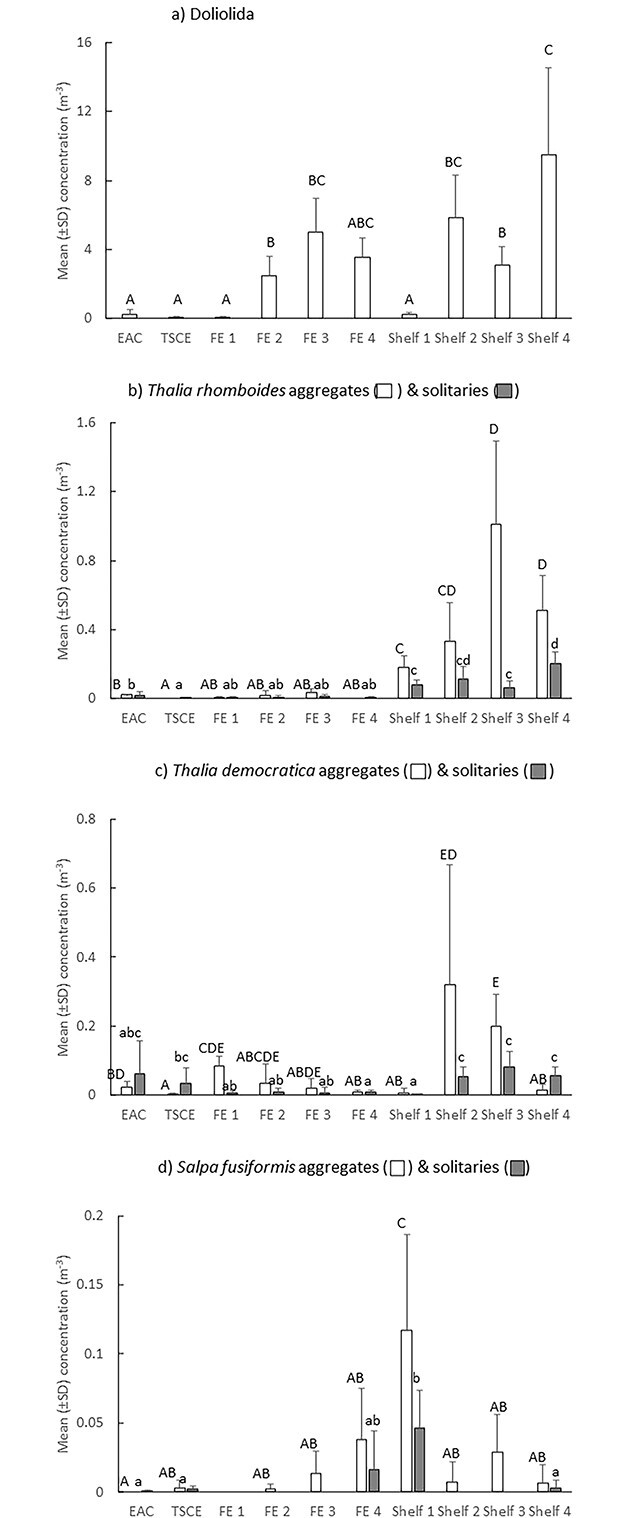
Autumn 2021. Mean (±SD) concentrations of Doliolida (**a**), and aggregate and solitary stages of *Thalia rhomboides* (**b**), *T. democratica* (**c**) and *S. fusiformis* (**d**) among oceanographic features and stations. EAC = East Australia Current; TSCE = Tasman Sea cyclonic eddy; FE = frontal eddy. Letters above bars indicate similarity (e.g. AA) or differences (e.g. AB) between oceanographic features for aggregates (capital letters) and solitaries (lower case letters). Note different scales on *y*-axes.

Doliolida were scarce offshore in the EAC and TSCE (Fig. 6a). They were also scarce during the first times the two coastal features (i.e. the FE and shelf waters) were sampled. Abundances increased by an order of magnitude between the first and second times the FE and shelf were sampled and remained abundant but variable in both features. Aggregates and solitaries of the salp *T. rhomboides* were scarce offshore and in the FE but were abundant in shelf waters, particularly during the third- and fourth-times sampled (Fig. 6b). Aggregates of *T. rhomboides* in shelf waters were 2 to 10 times more abundant than solitaries. *Thalia democratica* occurred in small numbers in all features and stations but aggregates were most abundant in the shelf waters during the second and third times sampled (Fig. 6c). *Salpa fusiformis* were absent in the FE during the first time sampled but aggregates increased in abundance each time the FE was subsequently sampled (Fig. 6d). In shelf waters, aggregates of *S. fusiformis* were most abundant during the first time sampled (Fig. 6d). Solitaries were absent at three of the four stations in the FE and at two of the four shelf stations.

### Relationship between thaliaceans and eddies

**Fig. 7:**
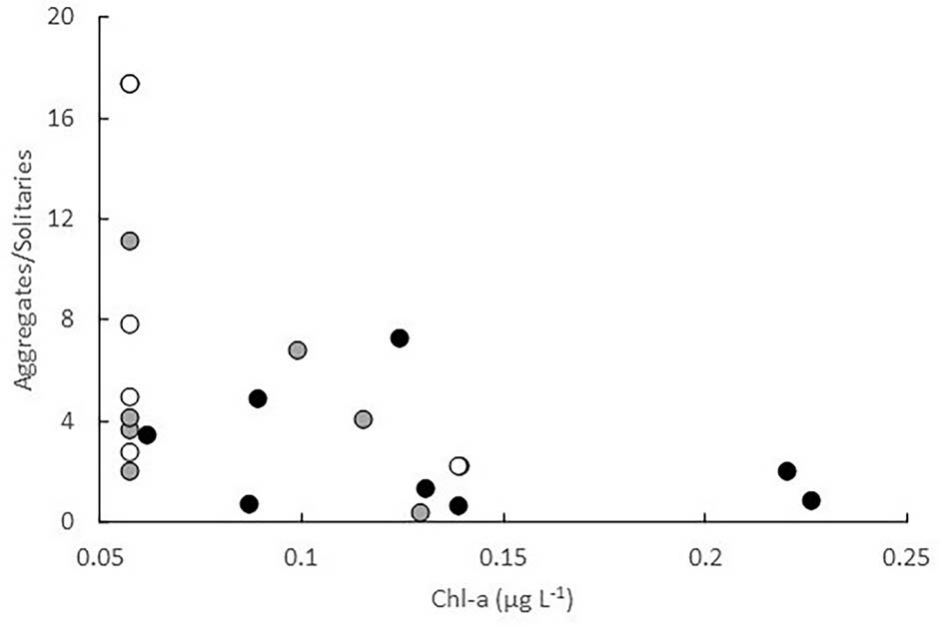
Relationship between *S. fusiformis* production (aggregates/solitaries) and chl-a concentrations in the LCE during the three times it was sampled in Spring 2019.

In Spring 2019, solitaries of *S. fusiformis* doubled and aggregates increased 6-fold over the 8 days the LCE was sampled (Fig. 4a). The average ratio of aggregates to solitaries of *S. fusiformis* (an index of production) increased from 2.6 (± 2.13 SD) to 10.3 (± 6.20 SD) between days 1 and 8, but ratios varied greatly among replicate tows. The production index for the LCE was inversely related to chl-a concentrations by the function Ln (Aggregates/Solitaries) = −0.7093 Ln Chl + 0.4095 (*r*^2^ = 0.3062; *P* = 0.005; Fig. 7). There was a trend for concentrations of *Pyrosoma* cf. *atlanticum* to quadruple over the same period but the trend was not significant (Fig. 4c). Aggregates and solitaries of *T. democratica* varied across the three times the eddy was sampled (Fig. 4b).

In Autumn 2021, very few thaliaceans occurred in the FE during the first time it was sampled (mean = 0.23 m^−3^  ± 0.06 SD). *Thalia rhomboides* and *T. democratica* remained scarce within the FE, despite being relatively abundant in nearby shelf waters that were sampled at similar times (Figs 6b, c). Aggregates of *S. fusiformis* increased in the FE through time and concentrations were comparable to the aggregates in the shelf waters during the last three times sampled (Fig. 6d). Solitaries of *S. fusiformis* were only observed in the FE during the final time sampled, which precluded calculation of a production index. Although Doliolida were initially rare in the FE, they increased > 50-fold and concentrations were comparable to those observed in shelf waters during the last three times sampled (Fig. 6a).

### Relationship between thaliaceans and chl-a

**Fig. 8:**
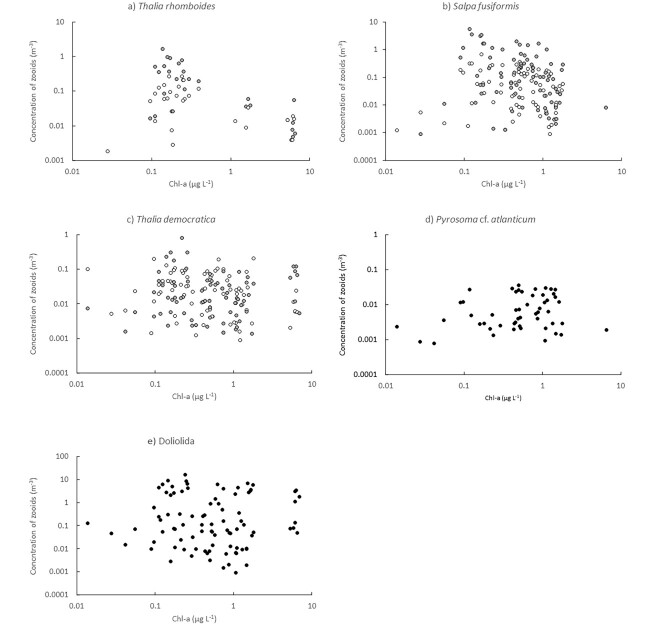
Relationship between concentrations of *Thalia rhomboides* (**a**), *S. fusiformis* (**b**), *T. democratica* (**c**), *Pyrosoma* cf *atlanticum* (**d**), and Doliolida (**e**) and chl-a. Solitaries (white symbols) and aggregates (gray symbols) are shown for salps.

Thaliaceans were most abundant when concentrations of chl-a were < 1 μg L^−1^ (Fig. 8). *Thalia rhomboides* were scarce (<0.1 m^−3^) when concentrations exceeded 0.4 μg L^−1^ (Fig. 8a). *Salpa fusiformis* was almost absent at the highest chl-a concentrations and was most abundant at concentrations of chl-a < 1 μg L^−1^ (Fig. 8b). *Thalia democratica* occurred in relatively small concentrations during both voyages but occurred in concentrations > 0.1 m^−3^ at the highest concentrations of chl-a (Fig. 8c). *Pyrosoma* cf. *atlanticum* occurred (with one exception) in concentrations of chl-a < 2 μg L^−1^ (Fig. 8d). Doliolida were the only thaliaceans that were relatively abundant (2–4 m^−3^) when concentrations of chl-a exceeded 5 μg L^−1^ (Fig. 8e).

### Variation in particle concentrations among oceanographic features

**Fig. 9:**
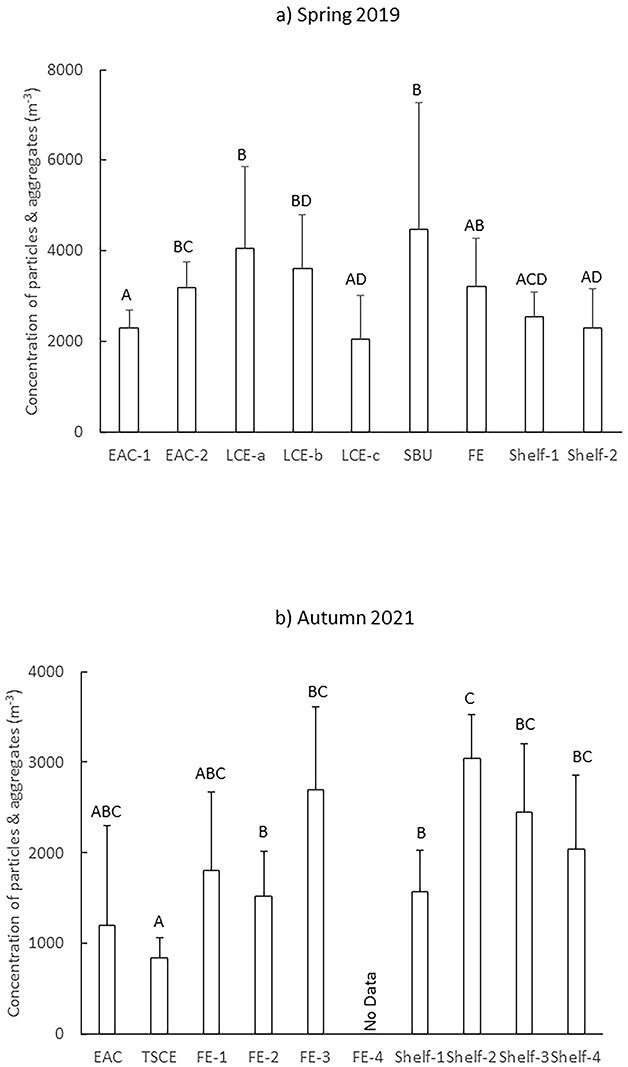
Mean (±SD) concentrations of particles and aggregates among oceanographic features and stations, counted using the LOPC during Spring 2019 (**a**) and Autumn 2021 (**b**). Note the difference in the *y*-axis scales. EAC = East Australia Current; LCE = large cyclonic eddy (sampled three times over 8 days; LCE-a, LCE-b, LCE-c); SBU = shelf break upwelling; FE = frontal eddy. Letters above bars indicate similarity (e.g. AA) or differences (e.g. AB) between oceanographic features.

Concentrations of particles, as measured by the LOPC, varied among features during Spring 2019 (Pseudo-F = 2.9906; *P* = 0.006) and Autumn 2021 (Pseudo-F = 3.8181; *P* = 0.003). In Spring 2019, concentrations were greatest at EAC-2, the shelf break upwelling, the small FE and the LCE during the first two times sampled (Fig. 9a). Notably, concentrations halved between the first and last time the LCE was sampled (Fig. 9a). In Autumn 2021, concentrations were lowest in the TSCE (Fig. 9b). Concentrations did not differ among the three times the 2021 FE was sampled but concentrations doubled between the first and second times the shelf waters were sampled.

## DISCUSSION

The distinctive mosaic of oceanographic features in the intensification zone of the EAC sampled in different seasons and years hosted diverse and varying communities of thaliaceans. Our results highlight the varying demographics and potential habitat preferences of different thaliacean taxa. A greater diversity of salps was observed in Spring 2019 than in Autumn 2021. This observation is consistent with thaliaceans generally being more abundant and diverse in Spring than in Autumn (e.g. Tew and Lo, 2005) but may also reflect the greater sampling effort (63 vs 39 tows), slightly deeper depths, and diversity of oceanographic features sampled in 2019. Each voyage was dominated by a single taxon; in 2019, *S. fusiformis* comprised approximately 66% of the catch, and in 2021, Doliolida were most common (87% of the total catch).

We hypothesized that cyclonic eddies would promote the production of thaliaceans, as has been observed in cyclonic eddies south of the separation zone of the EAC (Everett *et al.,* 2011; Henschke *et al.,* 2019). Although some thaliaceans did increase in abundance within the eddies, the responses varied among taxa. The LCE sampled in Spring 2019 contained the greatest concentration of salps found during that voyage. This eddy was mature and had moved well offshore when first sampled and abundances of aggregates of *S. fusiformis* increased > 6-fold within the eddy over the 8 days it was sampled. Concentrations of *T. democratica*, which was the second most abundant species sampled in 2019, however, remained small (<0.05 m^−3^). In contrast, the FE sampled four times in 2021 contained less salps than nearby shelf waters and the three most common salps exhibited different trends; *Salpa fusiformis* increased, *T. democratica* decreased and *Thalia rhomboides* remained scarce (<0.04 m^−3^). Doliolida were also rare (<0.1 m^−3^) within the FE during the first time it was sampled but abundances increased, and they were an order-of-magnitude more concentrated than salps during the last three sampling times (~2.5–5 m^−3^).

The 2019 and 2021 eddies exhibited extremely different chlorophyll dynamics. Despite being mature when first sampled, the LCE in 2019 initially contained similar or marginally lower concentrations of chlorophyll than other nearby oceanographic features and chlorophyll concentrations halved over the 8 days the eddy was sampled. In contrast, concentrations of chlorophyll in the FE sampled in 2021 were initially extremely high (>5 μg L^−1^), and although they decreased, concentrations remained > 5-fold higher than the adjoining shelf waters. The varying trajectories of thaliacean taxa within the eddies may reflect taxon-specific tolerances to particle loads.

All thaliaceans collect prey by filtering large volumes of water across fine mucous meshes that are secreted by the animal and then rolled into cords by cilia and ingested. Although thaliaceans were once considered to select prey based solely on size, we now know they can select among prey based on shape, surface properties and through complex behaviors that can reject food or prevent its ingestion (Conley *et al.,* 2018). These properties may, therefore, confer species with differing tolerances to particle loads. The 102 samples of thaliaceans we collected over the two voyages spanned two distinct chl-a concentration ranges; ~ 0.01–~ 2 and ~ 5–7 μg L^−1^, which enabled us to examine potential variations in tolerances of thaliaceans to chlorophyll loads. The salps *Thalia rhomboides* and *S. fusiformis* and the pyrosome, *Pyrosoma* cf. *atlanticum* exhibited the most restricted distributions and rarely occurred when concentrations of chl-a exceeded 0.4 μg L^−1^. *Thalia democratica* appeared to tolerate higher chlorophyll concentrations and occurred in low concentrations (0.1 m^−3^) even when chl-a exceeded 5 μg L^−1^.

Doliolida appeared more tolerant to high particle loads than salps and were relatively abundant (>2 m^−3^) at the highest chl-a concentrations. The mechanisms used by doliolids to pump water across their mucous mesh differs to salps, which may influence their ability to manage high loads. Salps pump water by contracting muscular bands that drive water across the mesh while simultaneously propelling the animals forward, whereas doliolids pump water using cilia on the gill bars (Deibel and Paffenhofer, 1988). Doliolids may tolerate higher particle loads because they can arrest the gill cilia (Conley *et al.,* 2018). Pyrosomes use a mechanism similar to doliolids to pump water across their mesh (Deibel and Paffenhofer, 1988), but occurred primarily in offshore waters and so may rarely encounter high chl-a loads. Some doliolids and some salps can cease production of the mesh, which enables particles to flow unimpeded through the filter (Deibel and Paffenhofer, 1988). Moreover, doliolids, and some salps can also “backflush” the pharyngeal cavity to remove unwanted particles (Deibel and Paffenhofer, 1988). The different trends observed among species in response to chlorophyll loads may thus reflect species-specific differences in the ability to avoid or reject high particle concentrations.

The inverse relationship we observed between *S. fusiformis* and chlorophyll concentrations in the LCE in 2019 was similar to that observed off the Mejillones Peninsula, Chile (Pagés *et al.,* 2001). In that region, *S. fusiformis* only occurred at stations with intermediate concentrations of chl-a (~40–70 mg m^−2^ (when integrated over 100 m depth, this equates to ~ 0.4–0.7 μg L^−1^) and were absent in upwelling areas where chl-a concentrations were high (120–160 mg m^−2^). Potential clogging of the salps’ feeding structures at high particle loads was proposed to explain the relationship (Pagés *et al.,* 2001). Ingestion rates of *S. fusiformis* have also been observed to decrease, as concentrations of algal cells (*Phaedactylum tricornutum*) increased from 2 to 64 × 10^3^ mL^−1^ (equivalent to 0.5–16 μg chl L^−1^; Andersen, 1985). Our observations are remarkably consistent with those of Pagés et al. (2001), with populations of *S. fusiformis* thriving only when concentrations of chl-a were below ~ 0.7 μg L^−1^.

The increase in temperature and decrease in chl-a observed in the LCE in 2019 is consistent with warmer EAC water being entrained into the eddy. Salps are voracious consumers of phytoplankton (Madin and Deibel, 1998). When abundant, grazing rates can exceed the population growth of phytoplankton (Zeldis *et al.,* 1995) but intense filtration by *S. fusiformis* on phytoplankton is unlikely to explain the decrease in chl-a concentrations in this eddy. Using measured clearance rates of *S. fusiformis* (Andersen, 1985) and conservatively assuming large sizes for the solitaries (100 mm) and aggregates (10 mm) we sampled, the maximum amount of water the combined life stages in each m^3^ could have filtered in 24 hours was ~ 42 liters or only 4.2% of the water available. Grazing by non-gelatinous zooplankton may have depleted the phytoplankton but concentrations of zooplankton (as measured by the LOPC) decreased over time in the LCE, which is inconsistent with grazing promoting zooplankton production. Instead, the reduction in chl-a and zooplankton may reflect dilution as the eddy became flooded with EAC water as it decayed. Indeed, the SST of the eddy increased > 0.6°C over the 8 days sampled, consistent with the warmer EAC surface water impinging into the core of the eddy. The rate of production of *S. fusiformis*, however, may have exceeded the rate at which the population was diluted, thereby masking the effects of dilution on the salp.

The FE sampled in Autumn 2021 had very different properties to the LCE sampled in Spring 2019. The FE formed when the EAC intercepted K’gari and it tracked south along the edge of the continental shelf. The eddy was first sampled about 4 days after it formed and the water temperatures within the eddy were approximately 2°C cooler than the surrounding waters, indicative of strong upwelling. The high concentrations of chl-a during the first two times the eddy was sampled probably resulted from rapid primary production fuelled by upwelling of nutrient-rich deep water from the shelf break. The negative correlation between *S. fusiformis* and chl-a was consistent with that observed in the LCE in 2019. The absolute concentrations of *S. fusiformis*, however, remained small (<0.05 m^−3^), which may reflect that, despite chl-a concentrations decreasing over time, concentrations remained high enough to prevent a rapid population increase of the salp. In contrast to *S. fusiformis*, *T. democratica* populations decreased in the FE. Although we sometimes observed *T. democratica* persisting in low concentrations in high chlorophyll loads, very high particle loads may prevent populations from thriving. Indeed, the extreme populations reported in cold core eddies south of the EAC separation zone coincided with much lower chlorophyll concentrations (<1 μg mL^−1^; Everett *et al.,* 2011), although it is not possible to determine whether the lower chlorophyll concentrations reflected grazing by *T. democratica*. *Thalia rhomboides* remained scarce within the 2021 FE despite being the most abundant salp in nearby shelf waters, suggesting that conditions within the eddy were not conducive to this species. The ecology of *T. rhomboides* is poorly known. In Taiwan, it occurred in abundances similar to those we observed in both oceanic and neritic waters during summer and winter (Liao *et al.,* 2013). Although abundances of *T. rhomboides* were not correlated with chl-a in that study, concentrations of chl-a were consistently low (<0.5 μg L^−1^). *Thalia rhomboides* may also be unable to tolerate high concentrations of phytoplankton but feeding experiments would be required to confirm this hypothesis.

Doliolids can occur in both inshore and offshore waters (e.g. Benguela system; Martin *et al.,* 2017). In 2021 Doliolida were scarce offshore but abundant in the shelf waters and the FE. In 2019, Doliolida were scarce in all features except the small FE, which had entrained and transported shelf water offshore. These observations highlight the importance of frontal eddies and shelf waters in production of Doliolida in this region and are consistent with observations in Taiwan, where Liao et al. (2013) observed that doliolids were abundant in shelf and neritic waters and rare within the offshore Kuroshio current. They speculated that doliolids prefer waters that are cooler, less saline and have more chl-a than salps and that they avoid the turbulent and higher current velocities of the offshore Kuroshio current, and our observations are consistent with this interpretation.

In the western Atlantic Ocean, doliolids commonly bloom in shelf-break upwellings (Deibel and Paffenhofer, 2009). We sampled a shelf break upwelling in Spring 2019 but found very few Doliolida. The high rates of primary production in shelf-break upwellings are thought to promote production of doliolids but a seed population is needed for blooms to form (Deibel and Paffenhofer, 2009). Doliolids have a population doubling time of 3–4 days (Paffenhofer and Lee, 1987) but the shelf-break upwelling in 2019 had formed only 1–2 days before it was sampled, which was probably insufficient time for the small seed population of Doliolida to increase.

The EAC jet supported fewest thaliaceans during both voyages but was distinguished in 2019 by containing 2–10 times more colonies of *Pyrosoma* cf. *atlanticum* than the other oceanographic features. Numbers of pyrosome colonies in the EAC jet exceeded, by an order of magnitude, those reported by Henschke et al. (2019) in a cold core eddy south of the EAC separation zone. The colonies we sampled, however, were much smaller (most < 20 mm vs mean of 107 mm) and the overall biomass of pyrosomes would have been much smaller than those reported by Henschke et al. (2019). Like other thaliaceans, pyrosomes are efficient phytoplankton grazers, consuming up to 95% of the daily phytoplankton standing stock (Henschke *et al.,* 2019). When phytoplankton are abundant (e.g. chl-a *~* 0.4–1 μg L^−1^), efficient grazing promotes extreme growth rates, equivalent to 30% body C d^−1^; (Andersen and Sardou, 1994; Henschke *et al.,* 2019). Given the relatively low concentrations of chl-a observed across most locations in our study, growth of pyrosome colonies in the EAC jet, may have been limited by the availability of phytoplankton.

Thaliaceans vary in their depth distributions and some undertake diel vertical migration (DVM). By sampling the sub-surface waters, we only assessed distributions of shallow-water taxa and those that migrated to surface waters at night. Pyrosomes undertake extensive migrations, the extent of which depend on the size of the colony, but all size classes accumulate in shallow (0–75 m) waters during the night (Andersen and Sardou, 1994). Doliolida (Eden *et al.,* 2009) and the salp *T. democratica* (Gibbons, 1997) are abundant in surface waters and exhibit either limited or no DVM. *Salpa fusiformis*, however, often undertakes DVM but the extent of the migration can vary (Pascual *et al.,* 2017). Indeed, oceanographic features, such as eddies, can influence the extent of DVM in zooplankton generally (Eden *et al.,* 2009) and it is possible that some of the variation we observed among oceanographic features could have reflected variation in the extent of DVM.

## CONCLUSION

The mesoscale oceanographic features of the intensification zone of the EAC supported diverse and differing thaliacean communities, although overall abundances of thaliaceans were much lower than sometimes observed in the cyclonic eddies of the EAC separation zone (Everett *et al.,* 2011; Henschke *et al.,* 2019). Cyclonic (upwelling) eddies promote production of some thaliaceans but individual species respond very differently within eddies, potentially due to species-specific variation in tolerances to particle loads and comparative experiments are needed to confirm this. Our observations across two voyages and seasons support suggestions that salps and Doliolida are abundant in neritic waters but that salps are adapted to less productive environments than Doliolida (Gibson and Paffenhofer, 2002; Liao *et al.,* 2013). Understanding the dynamics of thaliaceans is important, given they are now recognized as both important prey (Hays *et al.,* 2018) and consumers in marine food webs and may be responsible for almost 30% of the carbon transported to the deep sea (Luo *et al.,* 2020).

## Supplementary Material

Supp_Fig_1_fbad024

Supp_Fig_2_fbad024

Table_Supp_1_fbad024

## Data Availability

Data are available from the senior author upon request.
