# Supplementary figures and images for: Assemblages of pelagic thaliaceans in oceanographic features at the tropical-temperate transition zone of a western boundary current
Source: J Plankton Res. 2023 Jun 2;45(4):677–92. doi: 10.1093/plankt/fbad024 (PMC10361811; doi:10.1093/plankt/fbad024)

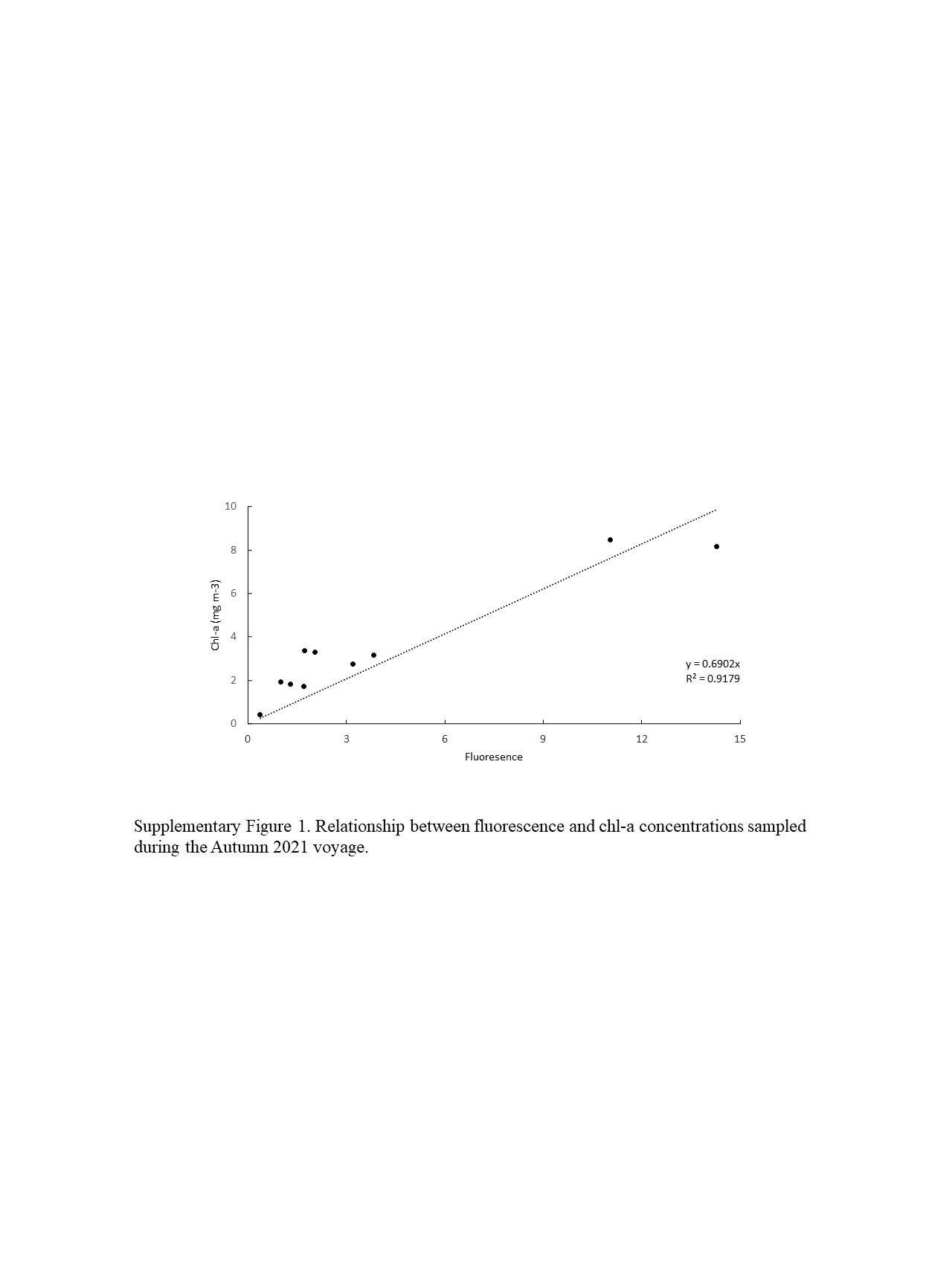

Supplement: Supp_Fig_1_fbad024 [file supp_fig_1_fbad024.jpeg]

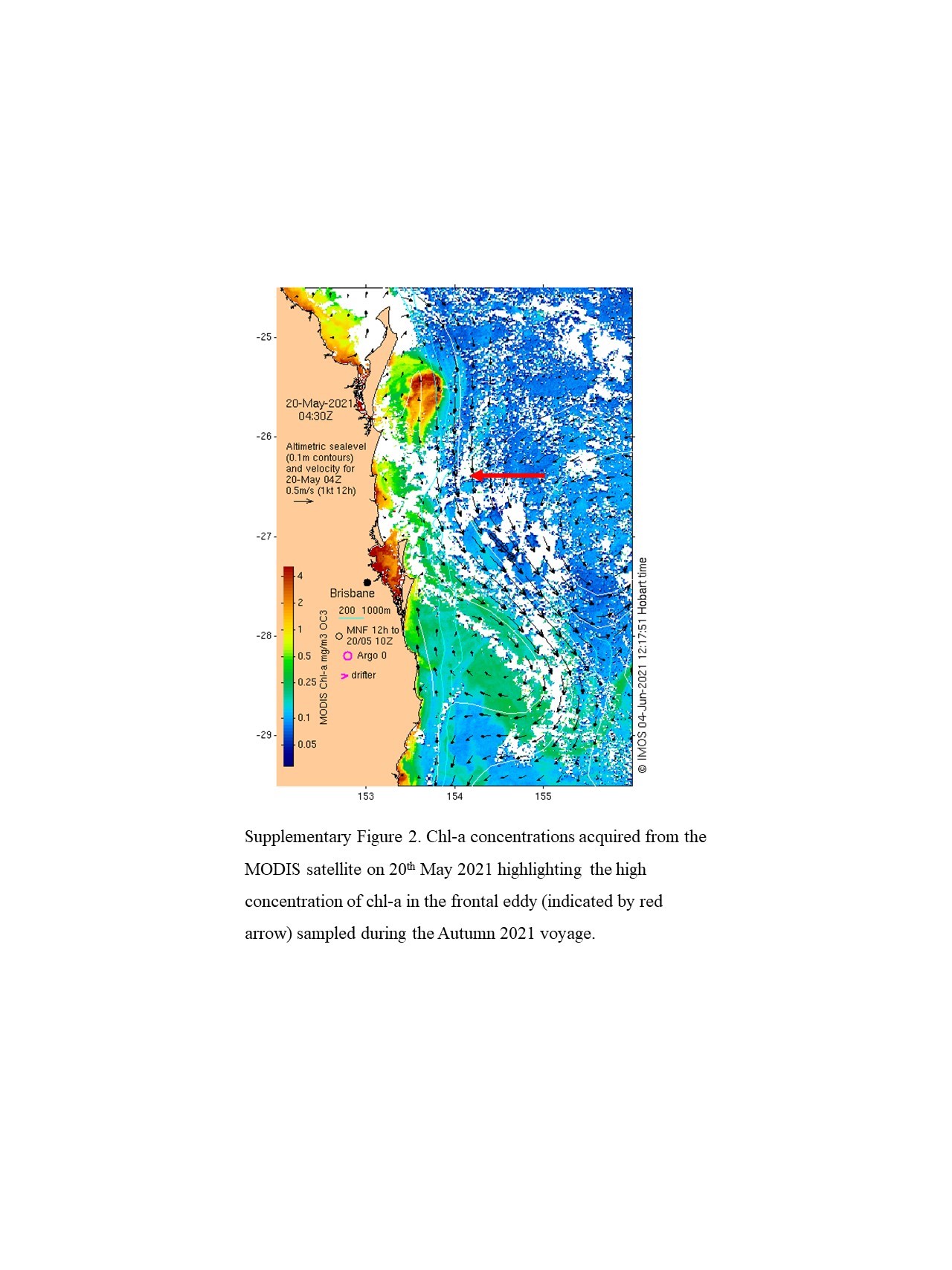

Supplement: Supp_Fig_2_fbad024 [file supp_fig_2_fbad024.jpeg]
