# Supplementary material for: Assemblages of pelagic thaliaceans in oceanographic features at the tropical-temperate transition zone of a western boundary current
Source: J Plankton Res. 2023 Jun 2;45(4):677–92. doi: 10.1093/plankt/fbad024 (PMC10361811; doi:10.1093/plankt/fbad024)
Supplement: Table_Supp_1_fbad024 [file table_supp_1_fbad024.docx]

Supplementary Table 1. Percentage contribution (%) of thaliaceans caught during Spring 2019 and Autumn 2020 voyages. Pyrosomes were counted as colonies but Doliolida and salps were counted as individual zooids. Sampling effort was greater in 2019 (63 vs 39 tows).

| Taxon | Spring 2019 (%) | Autumn 2021 (%) |
| --- | --- | --- |
| *Pyrosoma atlanticum* | <1 | <1 |
| Order Doliolida | 25.8 | 87.2 |
| *Salpa fusiformis* | 66.7 | <1 |
| *Thalia rhomboides* | 0 | 7.8 |
| *Thalia democratica* | 4.5 | 3.1 |
| *Pegea confoederata* | <1 | <1 |
| *Cyclosalpa affinis* | <1 | 0 |
| *Cyclosalpa bakeri* | <1 | <1 |
| *Cyclosalpla sewelli* | <1 | 0 |
| *Cyclosalpa pinnata* | 0 | <1 |
| *Cyclosalpa polae* | <1 | 0 |
| *Traustedtia multitentaculata* | <1 | 0 |
| *Metacalfina hexagona* | <1 | 0 |
| *Ritteriella amboinensis* | <1 | 0 |
